# Designing a novel ELISA method based on *CagA, NapA* recombinant antigens to increase sensitivity and specificity of *Helicobacter pylori* whole cell antigen detection 

**Published:** 2018

**Authors:** Farideh Kamarehei, Alireza Khabiri, Massoud Saidijam, Meysam Soleimani, Mohammad Yousef Alikhani

**Affiliations:** 1 *Department of Microbiology, Faculty of Medicine, Hamadan University of Medical Sciences, Hamadan, Iran*; 2 *Diagnostic Biotechnology Unit, Research and Production Complex, Pasteur Institute of Iran, Tehran, Iran*; 3 *Research Center for Molecular Medicine, Hamadan University of Medical Sciences, Hamadan, Iran*; 4 *Department of Pharmaceutical Biotechnology, Hamadan University of Medical Sciences, Hamadan, Iran *

**Keywords:** CagA protein, Helicobacter pylori, Neutrophil activating protein A, Enzyme-linked immunosorbent assay

## Abstract

**Aim:**

In this research, we designed a direct Enzyme Linked Immunoassay method to detect *Helicobacter* *pylori* antigens in stool specimens.

**Background:**

*Helicobacter pylori* infection as the worldwide problem is related to many gastrointestinal disorders such as gastritis, gastric cancer, non-ulcer disease, peptic ulcer disease and duodenal ulcer.

**Methods:**

We produced and purified recombinant *CagA* and *NapA* antigens in *Escherichia* *coli* and extracted their antibodies from a panel of positive sera specimens. We designed a novel enzyme linked immunoassay direct method in combination with the whole cell for the qualitative and quantitative detection of *Helicobacter* *pylori* antigens in human stool. Assay performance was evaluated by histopathology staining and urease activity.

**Results:**

The sensitivity and specificity of assay was determined as 91.7 [95% confidence interval: 89.3–95.6%] and 93.1% [95% CI: 91.2–96.4%], respectively. Novel ELISA exhibits enhanced sensitivity and specificity of *Helicobacter pylori* detection in comparison with another commercially available kit.

**Conclusion:**

Combination of the recombinant antigens and whole cell of *Helicobacter* *pylori* in immunoassay designing is a new approach about early diagnosis, treatment and fallowing up of the *Helicobacter pylori* infected patients, especially in peptic cancer cases.

## Introduction

*Helicobacter pylori (H. pylori)* infection is one of the most important bacterial infections has involved about half of the population worldwide (1). Infection with this bacterium has been proved to be associated with gastrointestinal disorders such as dyspepsia, active and chronic gastritis, non-ulcer disease, peptic ulcer disease, gastric ulcer, duodenal ulcer and gastric adenocarcinoma (2). *H. pylori* infection is present in over 90% of duodenal ulcers, 80% of gastric ulcers, and 70% of gastritis. This infection is more common in developing countries compared to developed communities (3). The transmission route for *H. pylori* is currently unknown but believed to be transmitted by oral-oral or fecal-oral route (4).

The diagnostic methods of *H. pylori* infection have been developed along two lines including the Direct; invasive and Indirect but non-invasive methods. The Giemsa staining and silver staining as gold standard and immunohistochemistry (IHC), rapid urease test (RUT), culture, histologic staining, and PCR on biopsy materials are direct, invasive and costly methods. The most common non-invasive methods include the serological detection of specific antibodies to *H. pylori.* Due to the decreasing sensitivity of direct diagnostic tests, several indirect tests including antibody-based tests (serology and urine test) and stool antigen test (SAT) have been developed for diagnosis of *H. pylori*. Another direct method is the urea breath test (UBT), though non-invasive and highly sensitive and specific, requires expensive laboratory equipment and moderate radiation exposure (5). More recently, the use of Enzyme-linked immune sorbent assay (ELISA) to detect the *H. pylori* antigens has been used for diagnosis of active *H. pylori* infection, patient monitoring after eradication therapy or reinfection. Individuals infected with *H. pylori* develop stool antigens which correlated strongly with histologically confirmed *H. pylori* infection (6). 

Most of the immunoassay methods were designed to detect *H. pylori* antibodies in serum. Early in the course of active infection, IgM antibody levels are detectable followed by a rise of IgG and IgA antibodies which remain constantly high until infection is eliminated but do not segregate between active and non-active infection. Also the serological tests that detect *H. pylori* IgG antibodies could also lead to false-negatives and have low sensitivity (7). In these tests, reduction of antibody titer during *H. pylori* progression, are the main factors associated with the false recent diagnosis in the laboratory incidence assays. Variable immune responses between different subtypes and/or populations, suppression of the bacterial replication by some *H. pylori* infected individuals need longer time to pass the cut-off point and some others always stay below this point, resulting in false negative.

The *H. pylori* antigen immunoassay is a major method for the qualitative and quantitative detection of *H. pylori* antigens in human stool. The first SAT was introduced in 1997, the polyclonal SAT (Premier Platinum *HpSA*, Meridian Bioscience Inc., OH, United States) with a sensitivity and specificity of 88.8% and 94.5%, respectively for diagnosis of *H. pylori* in untreated patients and following up of treated patients. It was followed by a monoclonal test (Femtolab *H. pylori*, Connex, Germany) (8). 

Similar to UBT, the results of the SAT sensitivity is affected by disorders of the digestive tract, the presence of a bleeding ulcer, acid-suppressing drugs known as proton pump inhibitors (PPIs), bismuth subsalicylate (Pepto-Bismol) and antibiotics. But, fasting is not needed for SAT and, sometimes unaffected by PPI; therefore, SAT is more advantageous than UBT (5, 9, 10). The selection of appropriate antigens is very critical. In addition to immune-reactivity, these antigens should have conserved sequences to cover different genotypes of *H. pylori*. Among different *H. pylori* virulence factors, Cytotoxin-associated gene A (CagA) as oncoprotein can affect the host cell biological pathways, such as the gastric epithelium cell tight junction and change the cytoskeleton, affecting the proliferation and differentiation of cells, and also stimulating inflammatory responses. *CagA* divided into the Western-type *CagA* and East Asian-type *CagA*, by the repeat sequence Glu-Pro-Ile-Tyr-Ala (EPIYA) motifs at the N-terminus of the protein (11). Moreover, several studies indicated that the *CagA*-positive strains are directly associated with acute gastritis, gastric ulcer, and gastric cancer development (12). On the other hand, *NapA* protein is a conserved and immunegenic product of *H. pylori*, encoded by the *napA* gene. Some of the clinical applications of *NapA* are vaccine development, clinical diagnosis and drug development (13). Investigators made many efforts to eradicate and overcome the disease by preventing, diagnosis and treating *H. pylori* infection. To achieve a better success concerning the prevention and treatment outcomes, the diagnosis of infection is very critical. The diagnostic assays for *H. pylori* infection are designed by combining the recombinant and synthetic peptides of *H. pylori* antigens. Currently, enzyme immunoassays (EIAs) are the most common immunological diagnostic methods used as the screening tool in *H. pylori* detection. These methods are very cheap, comfortable, accessible and non-invasive than others. 

In this study, we used a combination of the recombinant antigens (*CagA*, *NapA*) and the whole cell of *H. pylori* to identify this infection that could be develop an internal immunoassay diagnostic method. 

## Methods

 This study is in practical science field that was performed in Hamadan University of Medical Science from 2016 to 2018.

**Cloning, expression and purification of** ***H. pylori*** ***CagA, NapA* proteins and whole cell preparation**

In this study, a large batch of recombinant *CagA*, *NapA* was produced and purified previously, for coating the microtiter plates. In addition, we prepared whole cell antigen from Iranian *H. pylori* strain (Accession NO. JX428781.1) with the density equivalent to 9 McFarland standard by 10 times freeze and defreeze in liquid nitrogen and 42 ^o ^C, respectively (14).

**Specimens**

Primary optimization of ELISAs was conducted using synthesized recombinant proteins as antigens and a panel of human sera of sero-positive *H. pylori* infected patients to isolate the *CagA*, *NapA* antibodies from serum by affinity chromatography using ProG Sepharose (Abcam, UK) and also Cyanogen bromide (CnBr) activated-Sepharose (Sigma-Aldrich, USA). The panel consists of a set of 45 plasma specimens from different donors. The members were characterized using IgG ELISA kit (Pishtaz Teb Co, Iran) (15).

Sample size: For surveying the designed ELISA method, fifty two stool (26 positive and 26 negative) specimens were collected.

Inclusion/Exclusion criteria: The stool specimens were taken from outpatients referred to the gastroenterological private clinics who endure endoscopy, without antibiotic and inhibitor proton pomp therapy between 2017 and 2018. 

Table1 show the clinical and demographic characteristics of the patients enrolled to this study for stool specimen collection. These specimens were taken from Iranian *H. pylori* infected individuals to evaluation of the specificity and susceptibility of the selected developed ELISA. The *H. pylori* presence or absence was characterized based on the consensus results from two diagnosis methods; Rapid Urea Test (RUT) and Giemsa staining of stomach biopsies. Informed consent was obtained from all individual participants included in this study and the study was issued on 12.03.2016 and was approved by the local ethics committee of the Hamadan University of Medical Science, Hamadan, Iran (IR.UMSHA.REC. 1394.488). 

Because without refrigeration, the SAT suffers a significant reduction of sensitivity within 2 to 3 days, but with frozen stool samples stored (−80°C) for up to 225 days, therefore; we took the stool samples and stored for 72 h at 4 °C (16). 

**Developing ELISAs targeting** ***CagA, NapA*** **and whole cell antigens**

Different concentrations of anti-*CagA*, *NapA* and whole cell separately were prepared in coating buffer (0.1 M carbonate-bicarbonate buffer, pH 9.6) added to 96-well microtiter plate (Nunc, Roskilde, Denmark) and left at room temperature (RT) overnight. The coated plates were washed 2 times with PBS and 0.05% Tween-20 (PBS-T) and blocked with 1% BSA in PBS for 1 h at RT. In all assays, the positive and negative controls were used. Whole cell and recombinant antibodies were conjugated to horseradish peroxidase enzyme (Sigma Aldrich, St. Louis, USA) by sodium periodate method. The specimens and HRP-conjugated reactants were diluted in PBS-T-BSA (PBS in 0.1% Tween 20 and 2% bovine serum albumin). In this study, different ELISAs were designed, each being described separately below (15).

**Table 1:** The clinical and demographic characteristics of the patients enrolled to this study for stool specimen collection

Characteristics		Number (%)
Gender		
	Male	32 (62)
	Female	20 (38)
Mean of age		56 years
Type of the disease		
	Dyspepsia	28 (54)
	Gastritis	18 (35)
	Cancer	6 (11)
Total		52(100)

**Two-step direct ELISA for detection of whole cell antigens of** ***H. pylori***

The microtiter plates were coated with different concentration of anti-whole cell (0.5, 1, 2, 2.5 and 5μg/ml) with constant whole cell antigen concentration (50ng/ml) and the different concentration of anti- whole cell -HRP conjugate (1/500, 1/1000, 1/1500, 1/2000, 1/2500, 1/3000, 1/3500, 1/4000). Direct ELISA was conducted in conventional format; 100μl of the antigen were added on the antibody coated wells for 1 h at 37 °C. After 4 washes with 400μl PBS-T, 100μl of diluted of anti- whole cell -HRP conjugate (Sigma Aldrich, St. Louis, USA) was added to each well and incubated at 37 °C for 1 h. Wells were again washed as above, followed by addition of 100μl TMB, and left for 15 min at RT. The enzymatic reaction was stopped by addition of 50μl of 0.5 M H2SO4 and the optical densities were measured at 450 nm. In the avidity format, after the first incubation (sample incubation and washing), the wells were incubated with 4 M urea solution for 15 min at 37 °C. After washing, the procedure was continued as in the conventional format (15).

**Two-step Direct ELISA for detection of** ***CagA*** **,** ***NapA*** **recombinant antigens of** ***H. pylori***

In this ELISA strategy was also designed in conventional format; the microtiter plates were coated with different concentration of each anti-*CagA* and *NapA* (0.5, 1, 2, 2.5 and 5μg/ml) with constant recombinant *CagA* and *NapA* antigen concentration (50ng/ml) and the different concentration of anti- *CagA* and *NapA* -HRP conjugate (1/500, 1/1000, 1/1500, 1/2000, 1/2500, 1/3000, 1/3500, 1/4000). The cross-reaction was surveyed and the rest of the procedure was continued as above (15).

**One-step Direct ELISA for detection of whole cell,** ***CagA*** **and** ***NapA*** **recombinant antigens against** ***H. pylori***

This step is similar to two-step direct ELISA, the difference is that antigen concentration is constant but the conjugated antibody was diluted in the antigen. We prepared double concentration of antigen (100ng/ μl) and also conjugated antibody but half of the amount of each (50 μl) was added. The procedure was continued as mentioned above. This procedure was done for whole cell, CagA, NapA with their antibodies and also their cross reactions were surveyed (15).

**Sandwich ELISA for detection of total and recombinant** ***CagA*** **,** ***NapA*** **antigens**

In this section, the recombinant antigens diluted in PBS as positive controls and diluted buffer as the negative control. They were added to each well of the microtiter plate, coated with the best concentration of *CagA*, NapA and whole cell and also mixed of the recombinant *CagA*, *NapA* antibodies; 0.5, 1.5 and 2μg/ml, respectively. After 1 h incubation at 37 °C and washing 4 times, 100μl of HRP-conjugated whole cell, *CagA*, *NapA* and *CagA*-*NapA* (1: 3000) was added to each well and the microplates were incubated for another 1 h in 37 °C. The procedure was continued by washing 4 times, adding TMB for 15 min and stopping the reaction by 50μl of 0.5 M H2SO4.In the avidity format, the antibodies, attached in the first step, were treated by adding 4 molar urea solution to each well and, then, the procedure continued as the conventional format (15).

**Analysis of the primary results with the sandwich Direct ELISA**

The tested were assayed in triplicate with different developed ELISAs; mean optical density of each well was calculated. As direct ELISA for detection of whole cell, *CagA*, *NapA* against *H. pylori* resulted in no significant signal; these tests were excluded from further analysis. For other assays, the best point for discrimination of samples was determined as cut-off point and this point was used for calculation of signal to cut-off ratio. The criteria for selection of assays, as optimized, were the condition resulting in the best discrimination of samples and negative control optical density of less than 0.1. Using these criteria, one format of each developed ELISA strategy was selected as the optimized assay. This optimized assay was compared using the chi-square test and the best one, the direct ELISA for detection of the whole cell antigens of *H. pylori*, was selected for further analysis (15).

**Evaluating the cut-off of assay**

All testes were assayed for 10 times, using microtiter plates coated by 1 μg/ml of the whole cell antigens of *H. pylori*. The cut-off point was calculated as mean OD plus 3 standard deviations. This cut-off point was used for evaluation of specificity of the assay (15).

**The pre-clinical studies of the developed immunoassay method**

For this step, 100μl of each stool specimens diluted in PBS-T-BSA, was added to the wells, coated by 1μg/ml of whole cell, and washed 4 times with PBS-T after 1 h incubation at 37 °C. Low avidity antibodies were dissociated by 15 min incubation of wells with 100μl of 4 M urea at 37 °C followed by washing 4 times. Then, 100μl of 1:3000 diluted HRP conjugated anti CagA-NapA antibody (Sigma Aldrich, St. Louis, USA) was added and incubated for 1 h at 37 °C. After washing 4 times, the enzymatic signal was developed by adding 100μl of TMB solution and the reaction was stopped by 50μl of 0.5 M H2SO4. Each sample was tested in triplicate and the mean of ODs was normalized, by dividing to the cut-off point, and used for further analysis.

**Comparison with the gold standard assay**

The Giemsa staining of the peptic biopsy is gold standard in *H. pylori* diagnosis. Also, RUT is one of the commercially available assays on the peptic biopsy. Subsequently, each results of the direct sandwich ELISA designed method was compared with two above tests due to analyze the clinical sensitivity and specificity of the designed immunoassay method.

**Comparison with ASTRA enzyme immunoassay**

The kit of *H. pylori* antigen in stool (Astra, Italy) based on whole cell antigen is another enzyme immunoassay that commercially available in our country. Our assay measures antigens against major variants of immune-dominant regions of *H. pylori*. The assay was performed following the manufacturer’s package insert.

**Determination of the analytical sensitivity of the developed assay**

The sensitivity of the developed ELISAs was determined by testing tenfold serial dilutions of 0.5 McFarland equivalence suspension of *H. pylori* culture ranging from 1.5×10^8 ^to 1.5×10^1 ^colony forming unit/mL and twofold serial dilutions of recombinant antibodies ranging from 1 ng/mL to 0.001ng/ml. Then, the ELISAs were run as described. In each plate, 20 wells were considered as blank and 100 µl of dilution buffer (1% BSA in PBS) was added to blank wells instead of antigen. The optical density of each well was read at 450 nm. The mean and standard deviation of 20 blank wells were determined. Then, the LOB (Limit of Blank) and the absorbance of LOD (Limit of Detection) were calculated for each assay using following formula (17-19).

*LOB = Mean *_Blank_ *+1.645(SD *_Blank_ *)*

*Ab LOD= Mean *_Blank_ *+3 SD *_Blank_

**Determination of the analytical specificity of the developed assays**

The specificity of the assays was determined by testing the supernatant of different bacterial strains mentioned in Table 2. The cultures with concentration equivalent to 1 McFarland were tested by designed ELISA, as described above. *E. coli* (PTCC: 1399) was used for further evaluation of possible cross reactivity of NapA of *H. pylori* designed ELISA.

**Statistical analysis**

Statistical analysis was performed using STATA version 12 software (Stata Corp LP, College Station, TX, USA). To compare the performance of different methods (Linearity-assay, Giemsa Staining and ASTRA-EIA), two independent proportion statistical test was performed and adjustment was used for multiple comparison tests. All of the statistical analyses were performed using SPSS 16.0 statistical software and the *P* values of less than 0.05 were regarded as statistically significant. 

## Results

**Cloning, expression and purification of** ***H. pylori*** ***CagA, NapA* proteins**

Previously, we produced and purified the recombinant CagA and NapA proteins. Briefly, for NapA all available coding sequences and aa records (19 nucleotide records and 63 aa records) of *H. pylori* strains was identified from NCBI using Mega software and a consensus sequence was extracted from all records. Also, the specificity of the primers was evaluated by Primer-BLAST. For *CagA*, the immune-reactive antigenic region of this protein was selected by ABCpred, Bcepred and Emboss antigenic web servers (20). For amplification, we used the genomic DNA of *H. pylori* strain circulating in Iran; RIGLD-HC180 (Accession NO. JX428781.1) (21). 

The coding genes was cloned into pET28a expression vector and transformed to BL21 (DE3) *E.coli*. The expression of recombinant proteins were induced by 1mM IPTG and the cells were harvested after 3 hours. The purification of recombinant proteins were done by Ni-NTA affinity chromatography under denaturing condition and its immunoreactivity was evaluated by western blotting; the details were mentioned previously (22). 

Previously, we described that the recombinant antigens were cloned into a prokaryotic T7 RNA polymerase expression vector (PET28A). We expressed and produced the recombinant C-terminal His 6 -tagged NapA and CagA and also whole cell. The molecular weight of the recombinant NapA-pET28a and CagA-pET28a expressed in *E.coli* was ~17 and ~40 KDa, respectively. The expressed recombinant antigens showed an immune-reactive as was revealed by SDS-PAGE and Western blot analysis using specific polyclonal antibodies of patient serums that obtaining approximately 92% purity. 

**Table 2:** Bacterial strains used to evaluate the specificity of designed ELISA for *H.pylori* detection

Bacterial Strain	Number	Bacterial Strain	Number
*Acinetobacter baumannii*	PTCC: 1797	*Klebsiella oxytoca*	PTCC: 1402
*Aeromonas hydrophila*		*Lactobacillus acidophilus*	PTCC: 1643
*Bacillus cereus*	PTCC: 1154	*Proteus mirabilis*	PTCC: 1776
*Bacteroides fragilis*		*Pseudomonas aeroginosa*	PTCC: 1310
*Enterobacter aerogenes*	PTCC: 1221	*Salmonella enterica*	PTCC: 1709
*Enterococcus faecalis*	PTCC: 1237	*Shigella dysenteriae*	PTCC: 1188
*Escherichia coli*	PTCC: 1395	*Yersinia enterocolitica*	PTCC: 1151
*Escherichia coli EPEC(M):O55:K 59 *	PTCC: 1269	*Escherichia coli (ETEC) *	PTCC: 1399

PTCC: Persian Type Culture Collection

**Antibodies extraction against specific antigens by affinity chromatography**

The *CagA*, *NapA* and whole cell antibodies need for designing immunoassay method extracted successfully by affinity chromatography using *ProG* and *CnBr* activated-Sepharose.

**Developing ELISAs targeting** ***H. pylori*** **antigens**

The best conditions leading to optimized concentrations among various Sandwich Conventional Direct-ELISA strategies were resulted as follow:

Each well of the microtiter plate was coated by 2µg/ml anti whole cell antigens. This designed method can detect 1µg/ml of *H. pylori* antigens in stool sample. Then we must add 0.5µg/ml and 1.5µg/ml anti conjugated *CagA* and *NapA*, respectively. Also, among different dilutions of the HRP conjugate, 1:3000 was best dilution.

In each format, the arbitrary cut-off point, resulted in the best discrimination was used for calculation of signal to cut-off ratio (S/Co). Since coated antibody is polyclonal and similar to conjugated antibody, therefore the epitopes might be capture and despite the dynamic feature of the solution not recognized by conjugated antibody. Therefore we used two step assays but there was no significant difference between one step and tow step assays. 

**Cut-off determination of the assay**

The mean OD value and standard deviation of repetitive Linearity-assay on was 0.231 and 0.035, respectively, and then the cut-off point of 0.336 (mean +3 SD) was used for evaluation of the infected patients.

**Analytical sensitivity and specificity of the developed ELISA assay**

Analytical sensitivity of the newly developed assay was determined by serial dilutions of whole cell of *H. pylori *strain and recombinant antibodies to construct a standard curve. The resulted equations were used to calculate the limit of detections. The final results of the sensitivity were evaluated. In addition, testing different bacterial strains that would be present in stool specimens (Table 2) with α-*CagA* and α-*NapA*-ELISA did not show signals higher than their limit of blanks (OD: 0.254 and OD: 0.123, respectively), indicating no cross reactivity with the tested bacteria. Testing the supernatant of *E. coli *culture grown under stimulation condition by α-*NapA*-ELISA resulted in mean optical density of 0.29, which was higher than the absorbance of the assay. The sensitivity, specificity, positive predictive value (PPV), negative predictive value (NPV) of assay was determined as 91.7 [95% confidence interval (CI): 89.3–95.6%] and 93.1% [95% CI: 91.2–96.4%], 92%, and 87%, respectively.

**Evaluation of the clinical specificity of the ELISA assay**

The stool specimens tested by the designed assay were evaluated with another commercially *H. pylori* detection ELISA kit (ASTRA, Italy). Although; there was not observed significant difference between them but we found lower sensitivity and specificity as 89.8% [95% confidence interval: 86.2–92.4%] and 83.5% [95% CI: 79.3–87.6%], respectively than our designed ELISA assay.

## Discussion

 More than the half of the world’s population involves *H. pylori* infection, especially in developing countries (1). However, few of the patients are affected gastric cancer, but the risk of gastric cancer in *H. pylori* infected patients is 3 to 6 fold higher than whose are not infected; therefore, it is strongly associated with gastric cancer (23). So, the screening and eradication of *H. pylori* for gastric cancer prediction is critical. Also, there is no relative biomarker for diagnosis of gastric cancer induced by *H. pylori *(23). 

The SAT enzyme immunoassay was used to detect the presence of antigens against *H. pylori* in stool samples. It is a reliable method to diagnose an active infection and also confirm effective treatment of infection or eradication (24-26).

The diagnostic accuracy of the SAT in detecting eradication of *H. pylori* infection has been evaluated (5). A recent study compared the ability of 2 SAT methods, the enzyme immunoassay (Premier Platinum *HpSA*) and immunochromatographic method (ImmunoCard *HpSA* STAT), to detect *H. pylori* after eradication therapy in dyspeptic patients. The sensitivity, specificity, PPV, NPV and accuracy were 100%, 91.0%, 84.6%, 100%, and 94.0%, respectively, for ImmunoCard *HpSA* STAT, and 84.9%, 92.5%, 84.8%, 92.5%, and 90.0%, respectively, for Premier Platinum *HpSA* that novel ELISA exhibits enhanced sensitivity and specificity of *Helicobacter pylori* detection in comparison with another commercially available kit.

The advantage of the monoclonal SAT compared to the polyclonal test, both for early diagnosis of infection and for validation of *H. pylori’s* eradication following treatment, has been reported. Another study evaluated 5 SATs, including 2 monoclonal EIAs and 3 rapid immunochromatographic assay tests, for their ability to diagnose *H. pylori* infection in adult patients with dyspeptic symptoms before eradication therapy. For the 2 EIAs, the sensitivity and specificity were 92.2% and 94.4%, respectively, for the Premier Platinum *HpSA* Plus test, and 48.9% and 88.9%, respectively, for the *H. pylori* antigen test. For the 3 rapid immunochromatographic assays, the sensitivity and specificity were 86.7% and 88.9%, respectively, for the One Step HpSA test; 68.9% and 92.6%, respectively, for the ImmunoCard STAT HpSA test; and 78.9% and 87%, respectively, for the *H. pylori* fecal antigen test. The Premier Platinum *HpSA* Plus EIA test was the most accurate test to diagnose *H. pylori* infection in adult dyspeptic patients (27). 

The sensitivity and specificity of the SAT vary in different clinical settings and whether the test is given pre- or post-treatment (6, 28). Therefore, a local test validation in order to find the best cut-off for each population, especially in populations with low *H. pylori* prevalence, may become very important (5).

In a study in 2007, Gill et al (29) developed an ELISA method of thermophilic helicase-dependent isothermal DNA amplification (tHDA) for detection of *H. pylori*. In their study they detected one antigen (UreC) among biopsy samples but in our study, we detected two specific antigens and also in stool samples that it is easy and comfortable for patients than biopsy sample and also however this method has high sensitivity and specificity than our study, they used probe, digoxigenin labeling and streptavidin but in our study we attempted to minimize the amount of material used and design a cost-economic method for *H. pylori* detection. 

The advantage of the monoclonal SAT compared to the polyclonal test, both for early diagnosis of infection and for validation of *H. pylori’s* eradication following treatment, has been reported (30). According to previous studies, detection of *H. pylori* antigen in stool using a monoclonal enzyme-linked immunosorbent assay is one of the most efficient non-invasive tests for diagnosis of infection in children (31). The *H. pylori* SAT seems to perform well in children, independent of the child’s age (32, 33). An analysis of 20 studies of the SAT in a total of 2789 pre-treatment patients for *H. pylori* revealed values for sensitivity, specificity, PPV, and NPV of 90%, 96%, 93%, and 93%, respectively. In 8 studies, a total of 307 children were examined for the validation of *H. pylori* eradication after treatment by the SAT, with a sensitivity of 97%, specificity of 97%, PPV of 88%, and NPV of 99% (34).

The whole cell of *H. pylori* is usually used in commercially available EIAs kits (ELISAs and rapid tests). Hence, the presence of cross reacting antibodies against these antigens leads to the appearance of repetitive false positive results in screening tests. Retesting the primary reactive samples with EIAs using other *H. pylori* antigens can considerably reduce the rate of false positive results. The products of *cagA*, *vacA* and *ureA* genes are frequently used as antigen in most EIA tests commercially available kits for diagnosing *H.pylori* infection. But, among all of the virulence genes of *H. pylori*, in this study, we preferred to use the products of *CagA*, *NapA *as recombinant antigens in combination with the whole cell of *H. pylori* to identify *H. pylori* infection that significantly improve the sensitivity and specifically of detection. The products of *cagA* and *napA* gene may act as an appropriate candidate in this context.

In several previous studies, the prevalence of the coding genes of virulence factors in various bacteria was reported (35-39). The selection of an appropriate antigen to study antibody responses for the diagnosis of the infection is very critical. In addition to immune-reactivity, this antigen should have a conserved sequence to cover different genotypes of *H. pylori*. This problem can be resolved by using antigens covering multiple subtypes or using conserved genes. As our target population was Iranian patients, the sequences of dominant subtype in Iran, were used. As it has been shown previously, there are only 3.47% differences between the sequence of *NapA* and the SWISS-MODEL (https://swissmodel.expasy.org) which is the most widely used reference strain of *H. pylori*. Most of the different residues are amino acids with similar physicochemical properties and hence percent similarity between these two sequences is 96.53%. Because of the high conservation of *napA* gene, the assays were optimized based on sera specimens from Iranian patients and subsequently evaluated with them. The possible advantage of this conserved antigen is that it may provide the opportunity to use the developed assay for other population infected by different *H. pylori* subtypes. 

The novel ELISA is useful to detect anti-*CagA* antibodies in East Asian countries, and the titer might be a marker for predicting chronic gastritis and also gastric cancer. East Asian-type *CagA* ELISA was comparable to that of conventional *CagA* ELISA. Some the studies reported that the sensitivity of two ELISAs differed depending on the *cagA *genotype. The sensitivity of East Asian-type *CagA* ELISA was higher for subjects infected with East Asian-type *cagA H. pylori* (*P* < 0.001), and the sensitivity of the conventional *CagA* ELISA was higher for subjects infected with Western *cagA H. pylori* (*P* = 0.056) (40).

In addition to high conservation and immune-reactivity, the selected antigen in this study has the advantage of this study and the performance analysis of the purified recombinant antigens showed good immune-reactivity. Western blot analysis proved that the recombinant *CagA* and *NapA* was specifically recognized by the sera of *H. pylori* infected patients. The cross reactivity of this assay was surveyed with the whole cell of each bacteria in stool specimen. There was the poor cross reactivity but not significant of the *NapA* antigen with the whole cell antigens of *E. coli*, because of the presence of this similar antigen in *E. coli* that cross reactivity may be caused.

Because of the importance of the *H. pylori *many researches were done in Iran (41, 42).

This study is evaluated two antigens for diagnosis of *H. pylori* infection in Iran. Although the test population is too small to come to a comprehensive conclusion, the preliminary results seem promising. The best way for measurement of *H. pylori* infection is monitoring infected individuals during a specific period of treatment time. In addition to high cost, this approach needs the long-term follow up of a very large population. Hence, cohort studies of *H. pylori* infection are proposed. The results obtained from the surveyed ELISAs were significantly different (*p*: 0.001). Therefore, we decided to choose direct ELISA for detection of *H. pylori*, as a simpler method, for further evaluation. Here, we used a cut-off point obtained by repeated tests. Although the number of samples used is not enough to select an absolute cut-off point, the preliminary results using this cut-off point showed that it is in acceptable agreement with the results of the commercial assays.

However, this cut-off point needs to be further revised by testing a large number of samples. Employing a calibrator for normalization of obtained optical densities in these assays leads to high reproducibility of the results and simplifying the comparison of the results between different runs and labs. 

Comparing the performance of the ELISA-assay with the two commercial tests showed that they are not statistically different. Encouraging results were achieved the specificity, positive predictive value (PPV), negative predictive value (NPV) of assay was determined as 91.7 [95% confidence interval: 89.3–95.6%] and 93.1% [95% CI: 91.2–96.4%], 92%, and 87%, respectively.

According to our results, the sensitivity and specificity of assay are 91.7 and 93.1%, respectively. The high specificity of the in the studied population may be due to the antigen used for this assay, covering any subtypes. The exploited antigen covers a wide range of *H. pylori*. As more than 95% of circulating recombinant form of *H. pylori* in Iran is dedicated and the remainder is rare. One of the major drawbacks of serologic assays for *H. pylori* is false negative of individuals with long term *H. pylori* infection. Applying serologic tests targeting the two different *H. pylori* antigens will increase the performance of the algorithms. The proposed assay in this article, due to the use of different antigen than other assays, may be a good option for employing this method in diagnostic assay. Appropriate characterization of the assay requires use of large number of patients to be able to make a statement. 

The *H. pylori* antigen EIA Kit is a solid phase enzyme immunoassay based on sandwich principle for the qualitative and quantitative detection of *H. pylori* antigen in human stool. It is intended aid in the diagnosis of possible *H. pylori* infection and in the follow-up of patients undergoing antimicrobial therapy.

The limitation of this study was accessibility of needed materials.

We suggest designing a quantitative direct ELISA method for *H. pylori *detection with this process.
